# The prognostic value of whole-genome DNA methylation in response to Leflunomide in patients with Rheumatoid Arthritis

**DOI:** 10.3389/fimmu.2023.1173187

**Published:** 2023-09-07

**Authors:** Yulan Chen, Qiao Wang, Haina Liu, Lei Jin, Xin Feng, Bingbing Dai, Meng Chen, Fangran Xin, Tingting Wei, Bingqing Bai, Zhijun Fan, Jiahui Li, Yuxin Yao, Ruobing Liao, Jintao Zhang, Xiangnan Jin, Lingyu Fu

**Affiliations:** ^1^ Department of Clinical Epidemiology and Evidence-Based Medicine, the First Affiliated Hospital, China Medical University, Shenyang, China; ^2^ Department of Rheumatology, The First Affiliated Hospital, China Medical University, Shenyang, China; ^3^ Department of Rheumatology, ShengJing Hospital Affiliated of China Medical University, Shenyang, China; ^4^ Department of Rheumatology, First Affiliated Hospital of Jinzhou Medical University, Jinzhou, China; ^5^ Department of Rheumatology and Immunology, Dalian Municipal Central Hospital, Dalian, China; ^6^ Department of Medical Record Management Center, the First Affiliated Hospital, China Medical University, Shenyang, China

**Keywords:** rheumatoid arthritis, Leflunomide, methylation, machine learning, prognostic

## Abstract

**Objective:**

Although Leflunomide (LEF) is effective in treating rheumatoid arthritis (RA), there are still a considerable number of patients who respond poorly to LEF treatment. Till date, few LEF efficacy-predicting biomarkers have been identified. Herein, we explored and developed a DNA methylation-based predictive model for LEF-treated RA patient prognosis.

**Methods:**

Two hundred forty-five RA patients were prospectively enrolled from four participating study centers. A whole-genome DNA methylation profiling was conducted to identify LEF-related response signatures via comparison of 40 samples using Illumina 850k methylation arrays. Furthermore, differentially methylated positions (DMPs) were validated in the 245 RA patients using a targeted bisulfite sequencing assay. Lastly, prognostic models were developed, which included clinical characteristics and DMPs scores, for the prediction of LEF treatment response using machine learning algorithms.

**Results:**

We recognized a seven-DMP signature consisting of cg17330251, cg19814518, cg20124410, cg21109666, cg22572476, cg23403192, and cg24432675, which was effective in predicting RA patient’s LEF response status. In the five machine learning algorithms, the support vector machine (SVM) algorithm provided the best predictive model, with the largest discriminative ability, accuracy, and stability. Lastly, the AUC of the complex model(the 7-DMP scores with the lymphocyte and the diagnostic age) was higher than the simple model (the seven-DMP signature, AUC:0.74 vs 0.73 in the test set).

**Conclusion:**

In conclusion, we constructed a prognostic model integrating a 7-DMP scores with the clinical patient profile to predict responses to LEF treatment. Our model will be able to effectively guide clinicians in determining whether a patient is LEF treatment sensitive or not.

## 1 Introduction

Rheumatoid arthritis (RA) is an antibody-mediated persistent inflammatory autoimmune disease that is characterized by inflammation and destruction of the synovial and bone tissues (1, 2). RA is estimated to affect ~ 1% of the global population, and can develop at any age (3). The prevalence of RA in China ranges from 0.32% to 0.36%, while the prevalence of RA in Liaoning Province, located in Northeast China, is 0.5%, ranking first in China (4). The prognosis of RA is poor, with many comorbidities, requiring lifelong treatment, causing significant economic burden to patients, family members, and society (5). Currently, there is no cure for RA internationally,among the optimal RA treatment strategies recommended by the European League Against Rheumatism (EULAR) are disease-modifying anti-rheumatic drugs (DMARDs), such as, Methotrexate (MTX) or Leflunomide (LEF) (6). If these drugs fail to satisfactorily treat disease when employed as a single-agent or in combination therapies, then other biologic DMARDs, including, TNF and interleukin-6 receptor (TNFi and IL-6Ri) inhibitors may be implemented (6).

In our country, the utilization rate of the LEF, second only to MTX, is 45.9% (7). Compared to MTX, LEF treatment is typically accompanied by relatively low incidences of serious side effects and compared to biological agents, LEF has a better cost-effectiveness As such, LEF would usually be attempted prior to biologic-based intervention (8), particularly in a developing country like China.LEF is the first DMARD that demonstrated a marked improvement in RA symptoms in the past decade (9). LEF activity is mediated by metaboliteteriflunomide, which interacts with nonspecific cytochrome P450 (CYP) and certain drugs inducers that undergo CYP2C9-mediated metabolic processing (10). Unfortunately, LEF treatment can also promote adverse events, namely, hepatotoxicity, gastrointestinal discomfort, headache, hypertension, peripheral neuropathy, and increased susceptibility to infection (11). As such, identifying biomarkers predictive of LEF responses is of significant value as a means of ensuring that patients can be administered personalized therapeutic regimens that are both safe and effective, thus, minimizing the risk of adverse treatment-related toxicity. In the past, the clinical predictors the erythrocyte sedimentation rate (ESR), C-reactive protein (CRP),Rheumatoid factor (RF) and anticyclic citrullinated peptide antibody (ACPA) are representative serological markers for RA prognosis (12).

As one of the main regulation modes in epigenetics, DNA methylation is reversible, that is, demethylation. It can be regulated by diet, drugs and other environmental factors, so it can be used as a good therapeutic candidate. Several prior investigations revealed that DNA methylation patterns serve as biomarkers for estimation of RA patient therapeutic responses. Glossop et al. (13) quantified DNA methylation patterns in T cells using HumanMethylation450 BeadChips, which lead to the identification of 21 cytosine-phosphate-guanines (CpGs) that, at initial diagnosis, were associated with early RA patient responses to DMARD treatment. Moreover, Nair et al. (14) conducted an epigenome-wide association analysis (EWAS) whereby they compared the differentially methylated positions (DMPs) of individuals who responded well to MTX treatment and those who did not (n=34 each). They revealed that four CpG residues were associated with marked improvements in disease activity, thus facilitating the development of personalized treatment for patients at an earlier stage of disease, based on their epigenetic profile. Whole blood DNA methylation signatures and overall increases in DNA methylation are linked to moderate reductions in 28-joint disease activity score (DAS28) values among MTX non-responders (15). In addition, Plantet et al. (16) evaluated the DNA methylation patterns among RA patients undergoing TNFi treatment. They identified two prominent DMPs belonging to the *LRPAP1* gene. Till date, however, no studies specifically assessed the value of DNA methylation patterns as biomarkers of LEF therapeutic response.

Herein, to explore DNA methylation predictor and develop a DNA methylation-based predictive model for LEF-treated RA patient prognosis, we utilized the 850k Illumina HumanMethylationEPIC Bead Chip as a tool for comparing LEF nonresponders versus responders after 6 months of LEF treatment in order to identify DMPs. Key DMPs were selected via Gene Ontology (GO) and Kyoto Encyclopedia of Genes and Genomes (KEGG) enrichment analyses, followed by the subsequent validation of these hub DMPs using a large-sample LEF-treated RA patient population. Subsequently, we developed a prognostic model capable of predicting RA patient responses to LEF treatment. Our prognostic model can potentially assist clinicians in making the best decision for personalized patient therapy and pharmaceutical intervention. It can also aid in determining RA patient prognosis.

## 2 Materials and methods

### 2.1 Study design and patient population

To conduct this prospective investigation with an ongoing 6-month follow-up period, 272 samples were collected from patients diagnosed with RA undergoing LEF therapy between June 2018 and June 2020 from four hospitals (the First Hospital of China Medical University, Shengjing Hospital of China Medical University, First Affiliated Hospital of Jinzhou Medical University, and Dalian Municipal Central Hospital) located in the central, southern, and western regions of the Liaoning Province. Of these, 21 patients were lost to follow-up, 4 patients dropped out of treatment due to adverse reactions, 2 patients had poor blood quality, and 245 patients were included in the final analysis.

In the study, patients who met the following inclusion criteria were enrolled: 1) patients were were older than 18; 2) They met the RA diagnostic criteria proposed by the American Rheumatology Society (ACR) and EULAR in 2010 (17). The exclusion criteria are below: 1) was pregnant or lactating; 2) was diagnosed with serious liver, heart, or kidney diseases or mental disorders or cancer; 3) was diagnosed with other rheumatoid disease (e.g gout, osteoarthritis); 4) was diagnosed with other autoimmune diseases (e.g systemic lupus erythematosus (SLE), inflammatory bowel disease, ankylosing spondylitis, and multiple sclerosis); 5) use other DMARDs or biologics instead of LEF alone. Finally, Prior to the LEF treatment, baseline blood samples and clinical data were acquired from all participants. All participants were provided with a recommended stable LEF dosage of 20 mg/day for 6 months (18, 19). This investigation followed the Declaration of Helsinki, and received ethical approval from the Medical Science Research Institute of the First Affiliated Hospital, China Medical University, with all patients providing written informed consent (approval number: AF-SOP-07-1.0-01).

All participant demographics and clinical information were acquired from the aforementioned four hospitals’ medical and laboratory records, including (1) Basic information: patient’s gender, age, and so on: (2) Behavioral factors: smoking behavior and alcohol usage, and so on; (3) Immune and biochemical indicators: rheumatoid factor (RF), cyclic citrulline peptide antibody (anti-CCP), erythrocyte sedimentation rate (ESR), and so on. Indicators were categorized, according to the standards of the inspection equipment used by the four medical centers, and normalized to ensure a standardized information entry. All serum indicator values were acquired from the electronic hospital information system of the four hospitals upon the patients’ first blood evaluation at admission prior to LEF consumption. Following 6 months of follow-up, indicators associated with the quantity of tender joints, quantity of swollen joints, visual analog scale(VAS), and ESR were acquired again for the calculation of DAS28 of RA patients.

DAS28 values were used to assess RA patient disease activity. Following LEF treatment for 6 months, patient response status was determined according to the current DAS28 values and improvements in these values (ΔDAS28 = DAS28baseline – DAS28current) as per EULAR criteria (20, 21). A good response was represented by a DAS28 improvement of > 1.2, with a final DAS28 score ≤ 3.2, while nonresponse was defined as an improvement in DAS28 ≤ 0.6 or 0.6 ≤ DAS28 ≤ 1.2, with a DAS28 > 5.1 score after LEF treatment for 6 months. Patients who were between the aforementioned criteria were classified as moderate responders. To enhance the statistical power, patients with a good or moderate response were combined into a group referred to as responders for differential methylation analyses. Among the 245 patients who underwent LEF treatment for 6 months, 96 were nonresponders, and 149 were responders, as per the EULAR criteria.

### 2.2 Illumina 850k beadChip analysis

Overall, 20 responders were randomly selected from 149 responders identified above, following which 20 age- and sex-matched nonresponders were selected for comparison purposes. A QIAamp DNA Blood Mini Kit (QIAGEN) was employed for DNA extraction from the collected blood samples, as per kit directions. Genome-wide methylation profiles were then defined using the Illumina HumanMethylationEPIC BeadChip (Illumina, Inc., CA, USA) (850K array), which provided a coverage spanning >850,000 CpG sites per sample throughout the genome.

### 2.3 Functional enrichment analysis

GO and KEGG enrichment analyses of the screened DMGs were conducted using the R clusterProfiler package (22). *P* < 0.05 was the significance threshold. And, a network of protein-protein interactions (PPI) was created using the Search Tool for the Retrieval of Interacting Genes/Proteins (STRING) to illustrate the correlation between notable DMG.

### 2.4 Replication using targeted bisulfite sequencing assay

Pyrosequencing was carried out for the verification of essential methylation variant variable methylation statuses to establish that the 850K array analysis were, in fact, true methylation differences, and not mere artifacts. The aforementioned DMPs were chosen for validation using the Targeted Bisulfite Sequencing (MethylTarget) conducted at the Genesky Biotechnologies Inc. (Shanghai, China). DNA samples were quantified using the fluorometric method, and DNA integrity was assessed via gel electrophoresis using a 1.3% agarose gel. Briefly, DMPs were analyzed, sequenced, then validated. Genomic DNA conversion employed bisulfite, which converted unmethylated cytosine residues to uracil residues, prior to Polymerase Chain Reaction (PCR) reaction, which, in turn, amplified the target DNA sequences. Product sequencing was then performed on an Illumina Hiseq seq (Illumina, CA, United States). Differential methylation status was compared at each position between responders (n=149) and nonresponders (n=96) using the t-test and logistic analysis, following which, methylated CpG sites (*P* < 0.05) were selected for additional analyses.

### 2.5 Data analysis

#### 2.5.1 850k BeadChip data analysis

Data preprocessing was done with the Chip Analysis Methylation Pipeline ChAMP package (version 2.14.0) from the R Studio (version 3.6.3) (23). Raw read data from IDAT files were converted to β values, defined as the ratio of methylated to unmethylated intensity for a given CpG residue (24). Probes were removed if they did not pass the detection cut-off as follows: (*P* < 0.01), associated with known Single Nucleotide Polymorphism (SNPs), associated with the X and Y chromosomes, or non-CpG probes. The calculated β values ranged from β = 0 (unmethylated) to β = 1 (fully methylated), and the beta-mixture quantile normalization (BMIQ) was then employed to decrease bias between the Type I and Type II probes (25). Subsequently, DMPs were identified by comparing the responders and nonresponders using the ChAMP package. Following normalization, a single value decomposition (SVD) analysis was used to identify batch effects, which were then eliminated with ComBat using the gene-wise linear models from the SVA package. DMPs with a *P* < 0.05 and a |Δβ| > 0.1 were selected for subsequent analyses. LEF-related differentially methylated genes (DMGs) were next identified, including, genes associated with both hypo- and hypermethylation. A DMP-based heatmap was constructed using the ComplexHeatmap (v1.6.0) and ggplot2 R packages (26).

#### 2.5.2 Prognostic CpG methylation-based model construction and validation

To assess the clinical basic characteristics and potential risk factors, we employed descriptive statistics. Continuous data, analyzed via the t-test, are presented as mean (SD), whereas, categorical data, analyzed via the χ2 test, are provided as frequencies and percentages. The main drug response confounders, such as the age, gender, smoking, Hypertension, Diabetes, alcohol-drinking and RF,Anti-CCP etc. were analyzed by t-test and χ2 test between responder group and non-responder group. Two-tailed probabilities were employed, and correlations were deemed when *P* values were <0.1. After that, the factors like the age at diagnosis,LY, MONO, and anti-CCP which were significantly different between two group would be put in the complex model.

CpG unites with missing values in over 20% samples were eliminated, as well as samples with missing values in over 20% of CpG unites. Subsequently, the entire dataset was arbitrarily separated into a training (156/245, 70%) and a test dataset (68/245, 30%). To achieve an enhanced prediction of prognostability of the selected CpGs bioindicators, five machine learning methods, namely, logistic regression, random forest (RF), support vector machine (SVM), adaboost, and naïve Bayes (NB) from the caret R package (version 6.0-86) were employed for the prognostic model generation for LEF response in RA patients, followed by the application of a 10-fold cross-validation (27). Based on an optimized cutoff value, the sensitivity, specificity, accuracy, F1-score, recall, and precision were obtained from the machine learning model to assess the methylation scores’ ability to differentiate between LEF responders and nonresponders. In the end, we conducted a predictive model incorporating these DMPs scores along with other clinical variables.

The Hosmer–Lemeshow test were employed for model calibration evaluation. The model predictive precision for individual endpoints (discriminating ability) was assessed via AUC. The net reclassification improvement (NRI) and integrated discrimination improvement (IDI) are two complementary validation methods that assess the enhanced predictive ability of a complex model compared to a simple model (28, 29). The NRI represented the patient population who were accurately restratified by the newly developed model, relative to the existing or standard model. The IDI referred to the alteration in difference between the mean estimated possibilities between the new and existing models. By calculating the NRI and IDI values of the simple model (the seven-DMP signature) and the complex model(the 7-DMP scores with other clinical variables), the differentiation ability of the models before and after the adjustment of clinical features was compared, and the optimal model was further figured out.

CpGs (Pearson’s correlation coefficient r > 0.5) with strong associations were eliminated, and a 10-fold cross-validation was conducted across all models. To maintain reproducibility, in all random sampling analyses, we adjusted the random number seed to 100 with the set.seed function prior to any random sampling.

## 3 Results

### 3.1 Cohort characteristics

The clinical criteria used for each indicator and patient characteristics analyzed in this investigation are summarized in **Supplementary Tables S1** and **S2**. At baseline, responder and nonresponder patients were between 57.54 and 58.10 years old, respectively, with no significant differences in baseline clinical parameters between the two groups. However, we did observe a significant difference in the age at diagnosis, LY, MONO, and anti-CCP between the responder and nonresponder groups (*P*<0.10)

### 3.2 Discovery epigenome-wide association assessment

A total of 3,478 probes were excluded from the analysis during the quality control and normalization process due to having a detection *P*-value greater than 0.01. In addition, 5,703 probes with a beadcount of less than 3 in at least 5% of the samples were removed. A further 2,970 probes were excluded from the analysis as they were not CpGs, and an additional 10 multi-hit probes were filtered out. Consequently, 16,590 probes located on chromosomes X and Y, as well as 92,002 SNP probes, were also excluded from further analyses. Overall, 740,429 CpG sites were screened for potential differences in methylation status between the responder and non-responder samples. Based on the SVD analyses, the marked differences between the two groups were attributable to the samples. According to the Slide or Array analyses, DMPs did not exhibit any significant variance (**Supplementary Figure S1A**).

**Figure 1:**
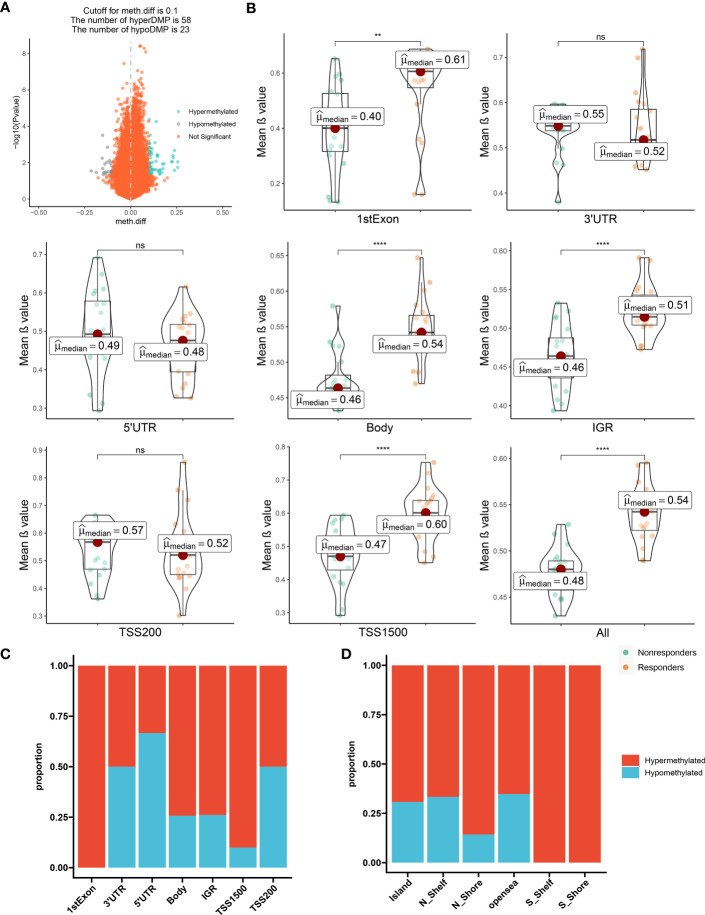
The distribution feature of DMCpGs. **(A)** Volcano plot of DMCpGs. **(B)** Comparisons of mean β value of DMCpGs by locations of CpG sites relative to gene structure. ns=not significant; * = *P* < 0.05; ** = *P* < 0.01; *** = *P* < 0.001; **** = *P* < 0.0001, by Wilcoxon tests. **(C)** Distribution of DMCpGs in relation to the gene region features. 1st Exon, the first exon of gene; 3’UTR = 3’-untranslated region; 5’UTR = 5’-untranslated region; IGR = intergenic region; TSS1500 = 1500nt upstream of TSS; TSS200 = 200nt upstream of TSS. **(D)** Distribution of DMCpGs in relation to the area related to CpG island. Island = CpG island; N_Shelf=2 kb upstream of corresponding N Shore; N_Shore=2 kb upstream of corresponding CpG island; opensea = the rest of the area; S_Shelf=2 kb downstream of corresponding S Shore; S Shore=2 kb downstream of corresponding CpG island.

These comparisons ultimately led to the identification of 81 DMPs (*P* < 0.05; |Δβ| > 0.1) between the responders to nonresponders, with 58 and 23 of these DMPs being hypermethylated and hypomethylated, respectively (**Figure 1A**; **Supplementary Figure S1B**).

Next, we mapped DMP distributions on human chromosomes using Manhattan plots, which revealed wide distribution across all non-sex chromosomes (**Supplementary Figure S1C**). Global methylation level comparison between these two patient subsets revealed that the mean β values of the CpG sites in the first Exon, body, Intergenic regions (IGR), and Transcription start sites(TSS1500) regions, as well as the overall methylation level, was significantly enhanced in responders versus nonresponders. Conversely, the methylation levels in the three prime untranslated region (3’-UTR), five prime untranslated region(5’-UTR), and TSS200 regions were augmented in the nonresponders versus responders, although the difference did not reach significance (**Figure 1B**). Consistently, the majority of CpG sites located in the first Exon, body, IGR, and TSS1500 are hypermethylated, but in contrast, the other regions showed the opposite trend. (**Figure 1C**). Moreover, the CpG sites within the Island, N_Shelf, N_Shore, opensea, S_Shelf, S Shore were more susceptible to hypermethylation (**Figure 1D**). Hence, in terms of epigenetics, we demonstrated a distinguishable methylation profile between responders and nonresponders, indicating that epigenetics potentially modulates LEF response.

### 3.3 Functional clustering analyses of significant DMGs

**Figure 2:**
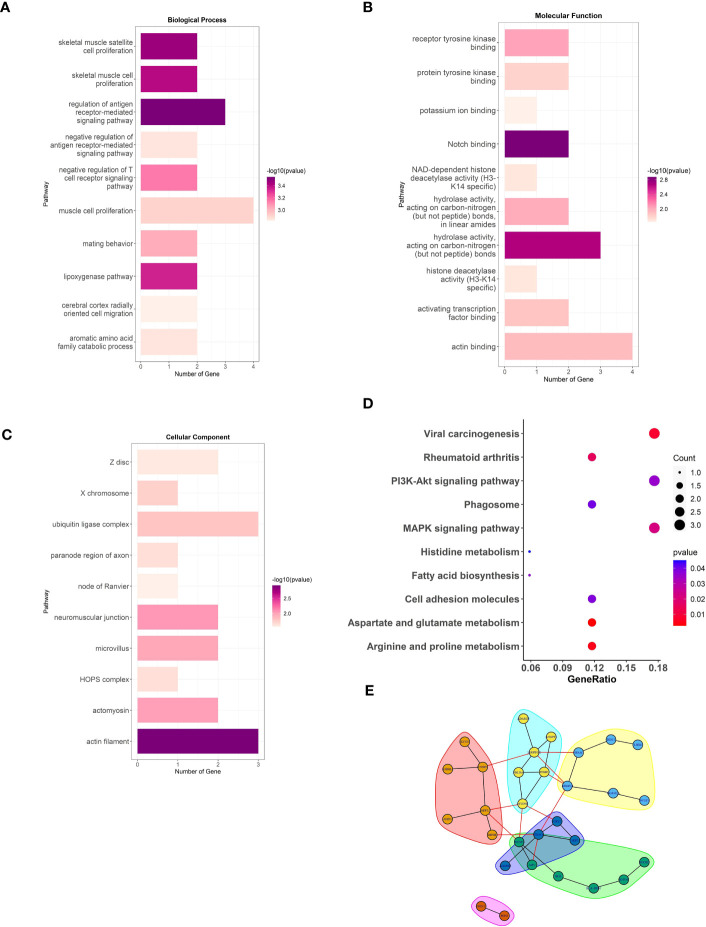
The analysis of DMGs. Three major GO enrichment analysis are shown in **(A)** biological process, **(B)** molecular function, and **(C)** cellular component, respectively. The right half-circle was the enriched GO terms, which were presented in different colors. The left half-circle was the gene enriched in these terms. **(D)**, KEGG pathways enriched by the DMGs associated with LEF response. The dot size represented the count of DMGs, and the color depth represented the *p*-value. **(E)**, The PPI network constructed with the DMGs.

To elucidate the potential physiological roles and signaling networks associated with the aforementioned DMGs, we conducted GO and KEGG pathway enrichment analyses (**Supplement Table S3**). Based on our analyses, the aforementioned DMGs were significantly enriched in GO terms associated with hydrolase activity, actin filament, muscle cell proliferation, and antigen receptor-mediated signaling pathway, as well as KEGG pathways, such as, viral carcinogenesis, rheumatoid arthritis, Pl3k-Akt signaling, and MAPK signaling (**Figures 2A–D**). These DMGs are thus likely to be associated with the RA-related metabolic processes. In order to investigate the interactions among the DMGs, a PPI network was constructed using STRING, a useful tool for evaluating the molecular functions of proteins. We identified 30 prominent proteins and selected six significant modules (**Figure 2E**).

### 3.4 Replication of epigenome-wide findings using Targeted Bisulfite Sequencing

We then performed bisulfite pyrosequencing (MethylTarget), another approach to evaluating DNA methylation, to confirm our earlier findings regarding the 81 DMPs. We validated seven DMPs between the 96 nonresponders and 149 responders. As expected, all seven DMPs exhibited consistent regulation within the samples, and the methylation values from MethylTarget were also consistent with the EPIC array (**Supplementary Figure S2**). The gene location and biological process (identified by the GO pathway enrichment analyses) of the seven aforementioned DMPs are summarized in **Table 1**. Based on our results, two hypomethylated DMPs (cg17330251, cg23403192) were located at the *PON1* and *USP16* promoters; another hypomethylated DMP (cg24432675) resided in the gene body of *ADARB2*; one hypermethylated DMP (cg19814518) was located at the *UHMK1* promoter; and one hypermethylated DMP (cg21109666) resided in the gene body of *DISC1*. Unfortunately, no corresponding genes were annotated to the other two hypermethylated DMPs (cg22572476 and cg20124410).

**Table 1:** the location and biological process of the validated methylated CpG sites.

CpG ID	Chromosome	Position	Gene	CpG Location	Type	Biological process	P.Value
cg17330251	7	94953956	PON1	TSS200-Island	Hypo	lipoxygenase pathway	0.030
cg24432675	10	1505472	ADARB2	Body-N_shore	Hypo	base conversion or substitution editing	0.006
cg23403192	21	30396327	USP16	TSS1500-N_Shore	Hypo	positive regulation of cellular amide metabolic process	0.039
cg19814518	1	162467080	UHMK1	TSS1500-N_Shore	Hyper	positive regulation of cellular amide metabolic process	0.002
cg21109666	1	232007841	DISC1	Body-opensea	Hyper	cerebral cortex radially oriented cell migration	0.005
cg22572476	6	28601324		IGR-N_Shore	Hyper	No found	0.002
cg20124410	13	107333224		IGR-opensea	Hyper	No found	0.008

### 3.5 Prognostic models development and validation

In light of the observed differences in DNA methylation between the LEF responders and non-responders at 6 months after baseline assessment, we sought to explore the predictability of DNA methylation profiles to estimate LEF therapeutic responses. As such, cg17330251, cg24432675, cg23403192, cg19814518, cg21109666, cg22572476, and cg20124410 were selected for use in machine learning models (a binomial model, 10-fold cross-validation) to generate a prognostic score.

**Figure 3:**
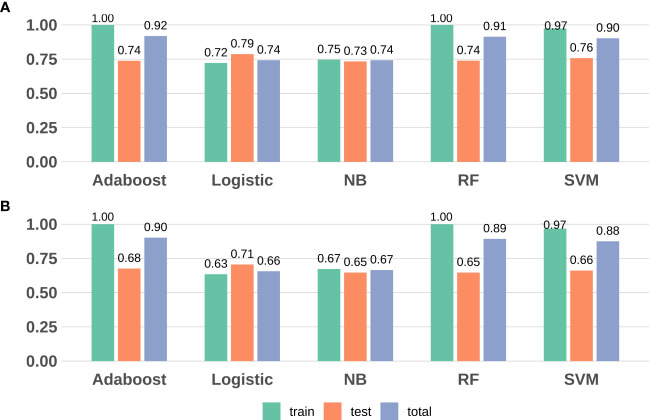
Model evaluation (F1-score and accuracy) results based on the seven DMPs across five machine learning. **(A)** F1-score; **(B)** Accuracy. RF, random forest; SVM, support vector machine; NB, naïve Bayes.

We revealed that, in the training data, all model sensitivities were between 0.79-1.00, whereas, specificities were between 0.40-1.00. The SVM model performed well in terms of accuracy, compared to the other machine learning models. In the Testing data, all model sensitivities and specificities were between 0.72-0.86 and 0.36-0.60, respectively. Thus, the SVM model again showed high precision, suggesting great strength and accuracy of the models. Moreover, we revealed that the prognostability was comparable between the training, testing, and the entire dataset in all models, indicating that our results were both robust and reliable (**Figure 3**; **Supplementary Table S4**).

### 3.6 Development of a prognostic classifier based on the methylation biomarkers and clinical factors

**Figure 4:**
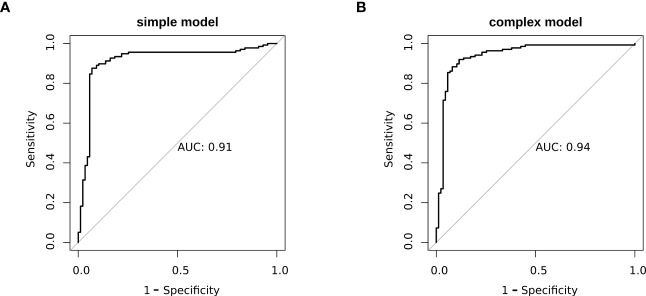
The ROC plots of models. **(A)** simple model **(B)** complex model.

To better elucidate the prognostic value of the selected bioindicators, we employed both methylation and patient clinical profiles to construct prognostic models for LEF response stratification. The age at diagnosis, LY, MONO, anti-CCP, were identified as independent predictors of patient response status (*P*< 0.1; **Supplementary Table S1**). The predicted values from the DNA methylation models, which serve as independent variables in the subsequent model, can be referred to as “7-DMP scores”. Since the 7-DMP scores were the strongest univariate predictors, we next generated the SVM models using the 7-DMP scores as well as each of the remaining significant clinical factors as complex models. All models based on the 7-DMPs (simple model) and any of the clinical signatures (complex model) exhibited similar predictive performance. However, the complex model based on 7-DMP scores, the age at diagnosis, and LY provided the most accurate predictions, confirming that these were independent predictive factors. The Hosmer–Lemeshow test exhibited no significant deviation from perfect fit in simple or complex models (*P >*0.05), suggesting good agreements between the estimated and actual outcomes. In the test set, we computed the AUC of the simple model (the seven-DMP signature) is 0.73, and the AUC of the complex model(the 7-DMP scores with clinical factors) is 0.74. The AUC revealed that the complex model (AUC=0.94) exhibited enhanced diagnostability, relative to the simple model (AUC=0.91) in the total set (**Figure 4**).

Based on our results of the NRI and IDI, both simple and complex models accurately restratified the RA patient’s response to LEF (**Table 2**). In summary, RA patients with reduced 7-DMPs scores, younger age at diagnosis, and negative LY experienced worse LEF responses, compared to their counterparts.

**Table 2:** Performance and internal validation of LEF nomogram.

	Simple model	Complex model
Hosmer-Lemeshow test		
χ²	1.97	1.99
*P*	0.85	0.86
Accuracy	0.875	0.9062
F1-score	0.9028	0.9231
Recall	0.9489	0.9197
Precision	0.8609	0.9265
AUC	0.91	0.94
NRI	ref.	0.09
IDI	ref.	0.09

AUC, area under the receiver operating characteristic curve; NRI, net reclassification index; IDI, integrated discrimination improvement; ref., reference level.

## 4 Discussion

This study employed Illumina 850k methylation arrays to assess DNA methylation profiles in whole blood samples from RA patients undergoing LEF treatment. We established a DMP-based prognostic signature that incorporated cg17330251, cg19814518, cg20124410, cg21109666, cg22572476, cg23403192, and cg24432675 that were sufficient to predict RA patient response to LEF following a 6-month treatment period. To our knowledge, this is the first study that identified LEF response-related DNA methylation biomarkers using Illumina 850k methylation arrays. This investigation provides a valuable foundation for efforts to support future personalized RA patient treatment.

Recently, LEF was recommended as a second-line DMARD following MTX by EULAR owing to its marked enhancement in functional disability and health-related quality of life (6). Clinical trials involving LEF demonstrated clearly that LEF treatment effectively reduced the signs and symptoms of disease, as well as radiographic progression (30, 31). However, LEF treatment withdrawal is a common challenge due to patient intolerance of adverse reactions, particularly upon the administration of loading doses (32). Prevention of LEF-induced undesirable effects still remains a challenging issue for the improvement of the clinical outcome of LEF-treated RA patients. As we mentioned before, in our study, DNA methylation was selected as biomarkers for predict the drug reaction because it is reversible and can be regulated by diet, drugs and other environmental factors, different from SNP and other gene mutations. Furthermore, another benefit of DNA methylation analysis is its ability to be conducted on non-invasive samples, such as blood, making it a more convenient and less invasive prognostic method than those requiring tissue biopsies (33).

Through analysis using an the 850k Illumina HumanMethylationEPIC Bead Chip, we identified 81 DMPs. According to Manhattan maps, DMPs sites were widely distributed on non-sex chromosomes. it suggests that DNA methylation alterations are not limited to specific genomic regions or chromosomes, but rather occur globally throughout the genome in RA patients. This implies that DNA methylation may play a widespread role in the development and progression of RA, and highlights the need for a comprehensive investigation of DNA methylation patterns in this disease. We observed that methylation levels of responders were higher in the promoter region such as the first Exon and TSS1500 than the non-responders. DNA methylation in promoter regions generally suppresses transcription or serves as a marker of a silenced gene (34). This is due to the strong binding of transcription factors or the recruitment of transcriptional repressors (35) and its dysregulation plays a crucial role in oncogenesis, tumor progression, and autoimmune diseases. Therefore, CpG islands located in promoter regions have always been of great interest to us, which is non-dynamic and less variant (36). Besides, methylation is more variable along the CpG shores, CpG shelves, and open sea (37). Therefore, it is more challenging and complicated to study methylation changes in CpG shores, CpG shelves and open sea in disease.

Based on our functional enrichment analyses of identified DMGs, we observed marked enrichments in GO terms, namely, hydrolase activity, actin filament, muscle cell proliferation, and the antigen receptor-mediated axis, all of which are closely related to RA progression (38–40). Based on the KEGG pathway analyses of these DMGs, the PI3K-Akt network was associated with key cellular functions, namely, survival, autophagy, differentiation, proliferation, and angiogenesis, and is a strong modulator of RA development and severity (41). Multiple reports suggested that MAPK signaling proteins control cellular responses to stressors and mitogenic stimuli, and that proinflammatory cytokines activate MAPK signaling in human fibroblast-like synoviocytes (FLS) in RA patients (42, 43). MAPK is therefore a critical modulator of RA progression, as well as a promising target for therapeutic intervention.

RA is generally marked with enhanced heterogeneity in patient prognosis, joint damage, and therapeutic response. For a majority of these factors, the mechanistic causality is yet undertermined (44). Therefore, currently, employing highthroughput technologies to develop computational methods to process patient -omic information to identify new and and precise conclusions is ever more important (45). For example, implementing machine learning algorithms in high-dimensional data analysis is a well-established approach to enhancing patient classification (46–48) or in predicting disease activity (49) in RA. Herein, we employed five machine learning approaches to analyze DNA methylation patterns, along with patient clinical information, to fine-tune the estimation of previously available stratifiers in an independent testing dataset.

**Figure 5:**
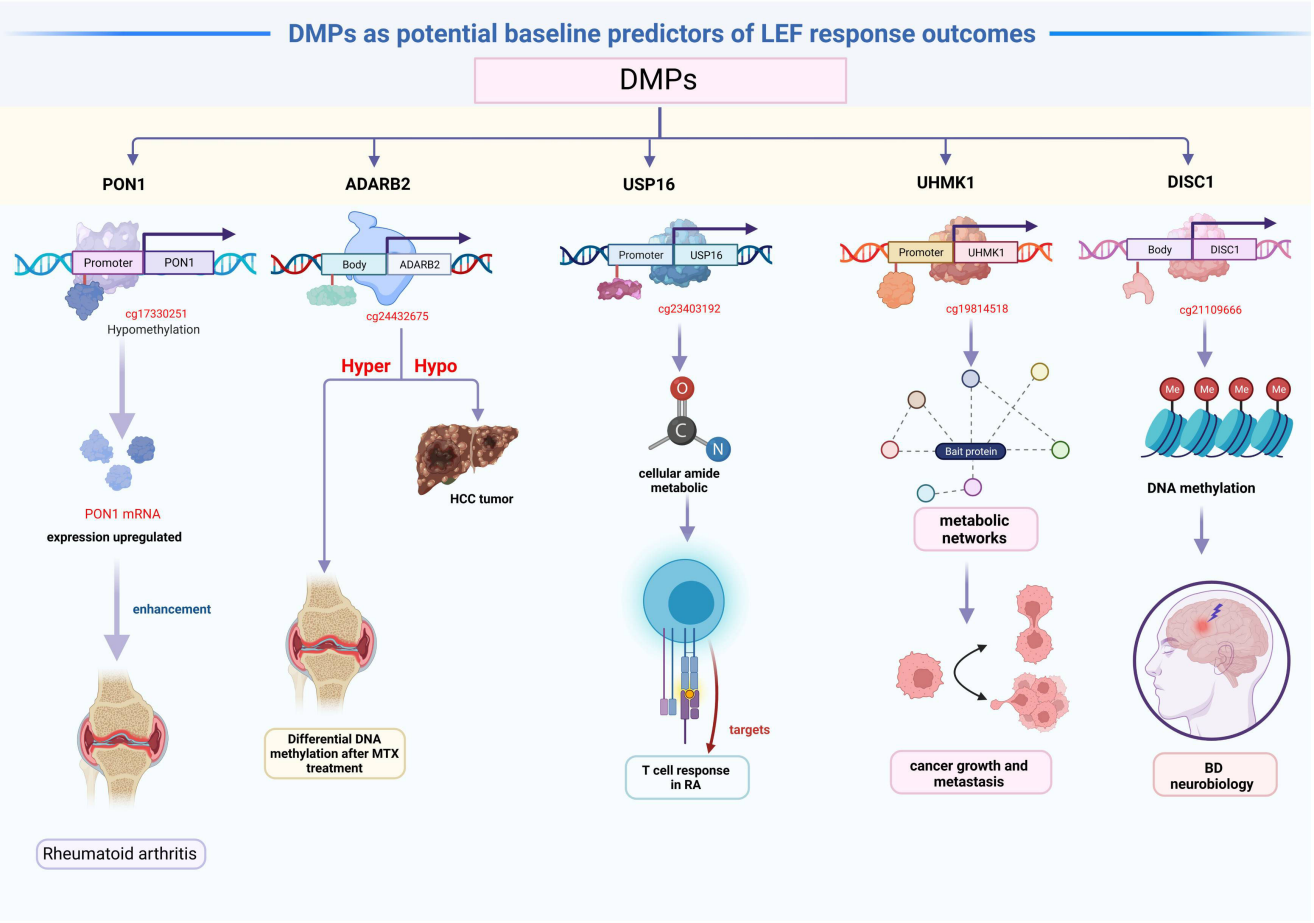
The mechanism figure of the main findings.

The 81 DMPs were chosen for validation using the pyrosequencing, which accurately detected differential CpG sites compared to the 850k methylation array. Our study explored suitable CpG biomarkers for RA response to LEF by both 850k DNA methylation microarray and a targeted bisulfite sequencing assay. After that, the 7 DMPs were identified to be predictors for RA response to LEF by developing prognostic models through several machine learning algorithms. Intriguingly, five of the seven DMPs were located at promoter sites or gene bodies of the annotated genes. Altered DNA methylation of genes, such as, *PON1*, *ADARB2*, *USP16*, *UHMK1*, and *DISC1*, was previously reported to be related to cancer or autoimmune diseases (**Figure 5**).

We identified hypomethylated cg17330251 located at the *PON1* promoter, and using GO analysis, we revealed that *PON1* was involved in the biological process of lipoxygenase pathway, which is a significant differentially modulated pathway in the GO categories. The lipoxygenase pathway may modulate eicosanoids generation, which, in turn, may impact cancer development, progression, and immune response (50). Additionally, the activation of the cyclooxygenase and lipoxygenase networks of arachidonic acid is speculated to contribute to rheumatic disease development (51). Therefore, we assumed that hypomethylated *PON1* promoters resulted in the high *PON1* mRNA expression, which, in turn enhanced the RA patient response to LEF treatment. A recent study suggested that *ADARB2* harbors the genetic MTX response variants, and demonstrated enhanced DNA methylation following MTX treatment in RA patients, compared to baseline (52). Similarly, the *ADARB2* gene body is extremely hypomethylated in HCC tumor, relative to adjacent tissues (53). This investigation suggested that the hypomethylated cg24432675 sites located at the *ADARB2* body may result in worse prognosis of LEF-treated RA patients. GO categories in both *USP16* and *UHMK1* were primarily related to the positive regulation of the cellular amide metabolic process, which may facilitate the metabolic regulation of the T cell response in RA (54). *USP16* is a critical deubiquitinase (DUB) for chromosomal segregation during mitosis (55). *USP16* also functions as a modulator of mature T cell activation, and may, therefore, serve as a new therapeutic target for T cell–mediated autoimmune disease treatment (56). In addition, *UHMK1* upregulation significantly promotes gastric cancer growth and metastasis possibly via metabolic networks (57). In contrast, impaired metabolism heavily modulates the origin and progression of autoimmune disease (58). We boldly speculated that the *USP16* and *UHMK1* may influence LEF response in RA patients via the metabolic network. *DISC1*, which encodes a scaffold protein, promotes hemoglobin synthesis in the peripheral blood (59). Several studies assessed the *DISC1* contents in PBMCs of BD patients. Based on their results, the DISC1 expression was correlated with an enhanced bipolar disorder (BD) risk, which may be due to DNA methylation, and it may be associated with BD neurobiology (60). In terms of the relevant DMPs in CD4 memory T cells, no studies till date, examined associations between their methylation status and RA patient responses to LEF treatment. Thus, further functional studies are warranted to validate these 7 DMPs role in modulating RA patients’ response to LEF.

In light of the above findings, we specifically constructed a biomarker signature, based on these seven candidates CpG sites, identified by the SVM model, We demonstrated that this signature was able to readily stratify patients into low- and high-risk response groups, with a great degree of accuracy (AUC=0.91). Furthermore, when the clinical data, such as, LY and age at diagnosis were included in this model, its predictive accuracy was further enhanced (AUC=0.94). Hence, we demonstrated that the simplified models with 7-DMPs was preferable since there was no significant improvement in NRI, relative to the complex model. Moreover, there was benefit owing to the use of less independent variables, which can reduce the burden on both doctors and patients, and minimize the overwhelming cost of medical diagnosis. However, in other cases of clinical usage, a medical doctor can opt for the complex model to minimize false positives, and gain accurate prognosis.

Our findings highlighted the convenience of synergistically employing both clinical and basic research information to obtain a complete and robust patient prognosis and therapeutic evaluation. In this study, the use of a prospective design in this study may have enabled the researchers to carefully screen and recruit participants, as well as gather data using standardized methods, thereby minimizing the likelihood of bias and confounding factors. No any other study had explored the DNA methylation as predictors for LEF-taking of RA patients, our conclusion highlights the potential of early treatment bioindicator monitoring in RA, and it raises the essential questions revolving prognosis of LEF responses. Assessing the efficiency of suitable stratification methods in the relevant context is crucial, as it can aid in predicting patient outcomes and improving therapeutic decision-making.

This study also had certain limitations. First, although our results from the 850k BeadChip array and targeted Bisulfite Sequencing Assay may be more representative and meaningful for future validation studies, futher experiments *in vitro* and *in vivo* should be performed to confirm and expand upon the aforementioned findings. Second, the short time of cohort analysis and the limited number of patients with DNA methylation data. Adequate follow-up time and multi-omics data in future studies would be valuable in further elucidating the prognostic significance of LEF response in RA patients. Third, although our samples were selected from four different hospital, all the four participating study centers were Third Class A hospical which may limit the generalizability of the findings to other populations or settings. The applicability of the identified DNA methylation profiles may be limited to the specific sample of patients enrolled, and replication of the findings in an independent sample is necessary to confirm their generalizability.

In the whole, the future work includes, on the one hand, conducting prospective studies in multiple clinical centers at different levels hospitals to perform prospective validation of our predictive model. On the other hand, conducting functional identification *in vivo* experiments at the cellular and animal models to determine the molecular mechanism underlying the results of this study

## 5 Conclusion

In summary, herein, we established a predictive prognosis model (the 7-DMP scores with the lymphocyte and the diagnostic age) to estimate RA patient LEF responses, and help to benefit RA patients’ LEF drug choice and nonresponders’ recognition. Our model showed much promise in guiding personalized patient treatment.

## Data availability statement

The datasets presented in this study can be found in online repositories. The names of the repository/repositories and accession number(s) can be found below: PRJNA946946 (SRA) and GSE228104 (GEO).

## Ethics statement

This investigation followed the Declaration of Helsinki, and received ethical approval from the Medical Science Research Institute of the First Affiliated Hospital, China Medical University, with all patients providing written informed consent (approval number: AF-SOP-07-1.0-01). The studies were conducted in accordance with the local legislation and institutional requirements. The participants provided their written informed consent to participate in this study.

## Author contributions

LF and YC conceived of the study. The data analysis was completed by YC, QW, HL, LJ, XF, BD, MC, FX, TW, BB and ZF, JL, YY, RL, JZ, XJ and LF helped to interpret the findings. The manuscript was written by LF and YC and content expertise was provided by all authors. All authors contributed to the article and approved the submitted version.

## Supplementary material

Supplementary Figure 1 **(A)** SVD plot of batch effects. **(B)** Heatmaps showing the top 81 CpGs from responders versus nonresponders to LEF. **(C)** Manhattan plots showing the −log10-transformed P values for CpG sites in the DNA methylation profiling for response to LEF.

Supplementary Figure 2 The relationship of the methylation values between from MethylTarget and the EPIC array.
