# Supplementary figures and images for: The prognostic value of whole-genome DNA methylation in response to Leflunomide in patients with Rheumatoid Arthritis
Source: Front Immunol. 2023 Sep 7;14:1173187. doi: 10.3389/fimmu.2023.1173187 (PMC10513488; doi:10.3389/fimmu.2023.1173187)

A

Singular Value Decomposition Analysis (SVD)

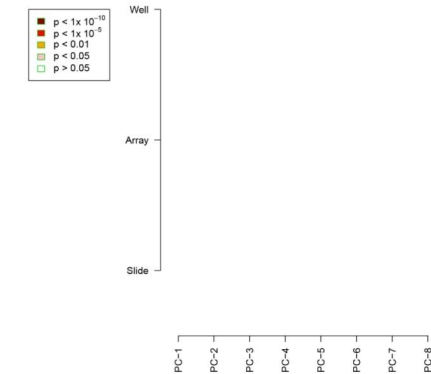

C

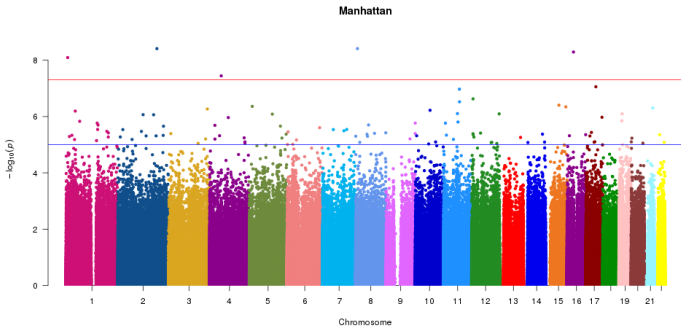

B

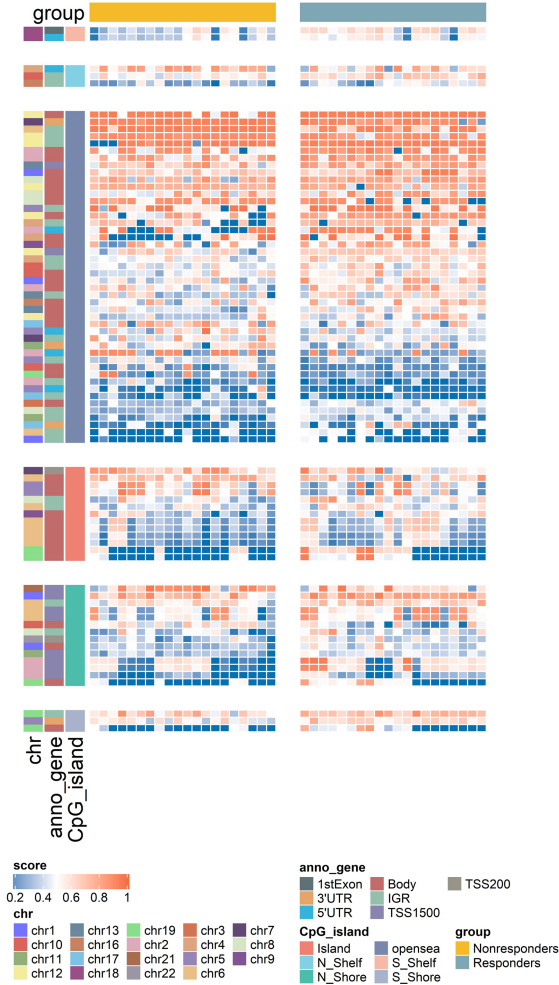

Supplement: Supplementary Figure 1 — (A) SVD plot of batch effects. (B) Heatmaps showing the top 81 CpGs from responders versus nonresponders to LEF. (C) Manhattan plots showing the −log10-transformed P values for CpG sites in the DNA methylation profiling for response to LEF. [file Image_1.pdf]

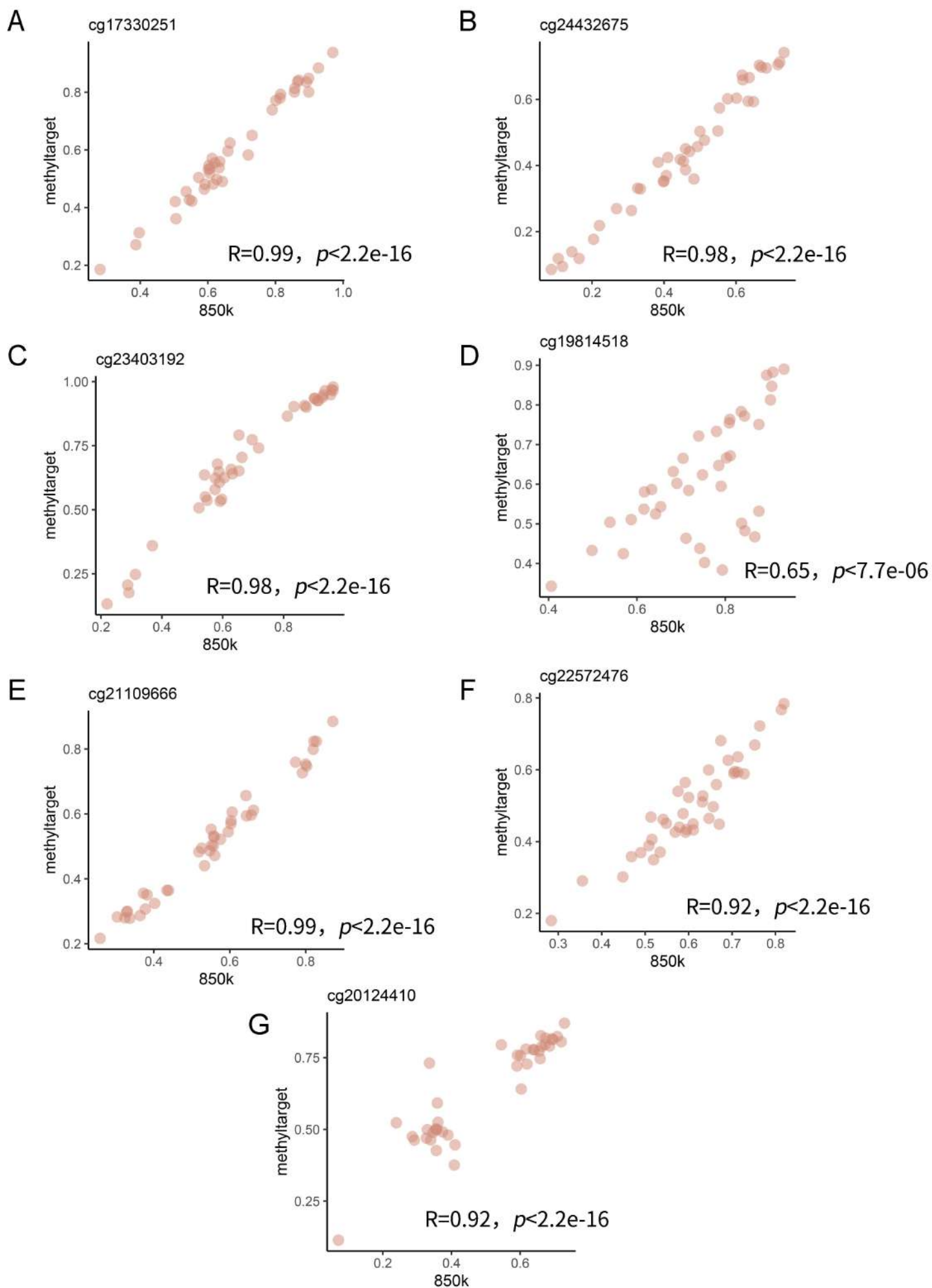

Supplement: Supplementary Figure 2 — The relationship of the methylation values between from MethylTarget and the EPIC array. [file Image_2.pdf]
