# Supplementary material for: The prognostic value of whole-genome DNA methylation in response to Leflunomide in patients with Rheumatoid Arthritis
Source: Front Immunol. 2023 Sep 7;14:1173187. doi: 10.3389/fimmu.2023.1173187 (PMC10513488; doi:10.3389/fimmu.2023.1173187)
Supplement: Supplementary file 3 [file Table_1.pdf]

Supplementary Table 1. The clinical criteria used for each indicator

	FACMU			SHCMU			FAJMU			DMCH		
	Negative	Medium	Positive	Negative	Medium	Positive	Negative	Medium	Positive	Negative	Medium	Positive
RF (IU/ml)	<=20	/	>20	<=20	/	>20	<=15.9	/	>15.9	<=20	/	>20
Anti-CCP (U/ml)	<=17	/	>17	<=20	/	>20	<=2.5	/	>2.5	<=5	/	>5
ESR (mm/h)	<=20	/	>20	<=20	/	>20	<=26	/	>26	<=30	/	>30
LY (*10^9/L)	<1.1	>=1.1,<=3.2	>3.2	<1.1	>=1.1,<=2.7	>2.7	<1.1	>=1.1,<=3.2	>3.2	<1.1	>=1.1,<=3.2	>3.2

FACMU, First Hospital of China Medical University; SHCMU, Shengjing Hospital of China Medical University; FAJMU, First Affiliated Hospital of Jinzhou Medical University; DMCH, Dalian Municipal Central Hospital; RA, rheumatoid arthritis; RF, rheumatoid factor; Anti-CCP, anti-cyclic citrullinated peptide antibody; ESR, erythrocyte sedimentation rate; LY, lymphocyte.
