# Supplementary material for: The prognostic value of whole-genome DNA methylation in response to Leflunomide in patients with Rheumatoid Arthritis
Source: Front Immunol. 2023 Sep 7;14:1173187. doi: 10.3389/fimmu.2023.1173187 (PMC10513488; doi:10.3389/fimmu.2023.1173187)
Supplement: Supplementary file 4 [file Table_2.pdf]

Supplementary Table 2. Descriptive and clinical variables of RA patients at baseline.

	level	Responders	Non-responders	p
n		149	96	
Gender(%)				0.627
	M	32	24	
	F	117	72	
Age (years) (mean (SD))		57.54(12.00)	58.10(13.54)	0.741
Age at diagnosis (years) (mean (SD))		53.05(12.96)	49.18 (13.91)	0.030
Diabetes (%)				0.644
	Yes	13	6	
	No	136	90	
Hypertension (%)				0.768
	Yes	20	15	
	No	129	81	
Smoking (%)				0.504
	Yes	19	16	
	No	130	80	
Alcohol.drinking (%)				0.331
	Yes	16	6	
	No	133	90	
LY				0.004
	Low	16	15	
	Medium	91	71	
	High	42	10	
MONO				0.040
	Negative	80	65	
	Positive	69	31	
RF (%)				0.957
	Negative	15	34	
	Positive	98	62	
Anti-CCP (%)				0.086
	Negative	42	38	
	Positive	107	58	
ESR (%)				0.507
	Negative	35	27	
	Positive	114	69	

RA, rheumatoid arthritis; SD, standard deviation; LY, lymphocyte; MONO, monocyte; RF, rheumatoid factor; Anti-CCP, anti-cyclic citrullinated peptide antibody; ESR, erythrocyte sedimentation rate. *P* value is derived by t-test for numeric variables and by chi-squared tests for categorical variables.
