# Supplementary material for: The prognostic value of whole-genome DNA methylation in response to Leflunomide in patients with Rheumatoid Arthritis
Source: Front Immunol. 2023 Sep 7;14:1173187. doi: 10.3389/fimmu.2023.1173187 (PMC10513488; doi:10.3389/fimmu.2023.1173187)
Supplement: Supplementary file 5 [file Table_3.pdf]

Supplementary Table 3. The list of DMP.

probe	CHR	UCSC_RefGene_Name	UCSC_RefGene_Group	Relation_to_UCSC_CpG_Island	meth.diff	P.Value	Type
cg12313947	8		IGR	opensea	0.101026472	4.2488E-05	Hypermethylated
cg18770216	3	ITGB5	Body	opensea	0.139054241	5.49986E-05	Hypermethylated
cg26125082	11	CRY2	TSS1500	N_Shore	0.10538062	0.000309754	Hypermethylated
cg11136343	5		IGR	opensea	-0.152847882	0.000312389	Hypomethylated
cg25463779	12	FAM101A	TSS1500	opensea	0.131578885	0.000790927	Hypermethylated
cg03873281	5	PDLIM4	3'UTR	S_Shore	0.100757791	0.000872112	Hypermethylated
cg12589188	10		IGR	N_Shelf	0.103498163	0.000881111	Hypermethylated
cg22445217	6		IGR	opensea	-0.126192355	0.001410012	Hypomethylated
cg13568171	1	MECR	Body	N_Shore	0.106202134	0.001494338	Hypermethylated
cg14804593	4		IGR	opensea	0.101650169	0.001550657	Hypermethylated
cg19814518	1	UHMK1	TSS1500	N_Shore	0.115925595	0.001751343	Hypermethylated
cg11258982	12	PAWR	Body	opensea	0.237363228	0.002130759	Hypermethylated
cg22572476	6		IGR	N_Shore	0.101894569	0.00238153	Hypermethylated
cg26825404	1	NFASC	Body	opensea	0.10717695	0.002631113	Hypermethylated

cg25065716	10	ADARB2	Body	opensea	0.111542094	0.002661012	Hypermethylated
cg17886420	10		IGR	opensea	0.112900656	0.003433511	Hypermethylated
cg17749961	2	LCLAT1	TSS1500	N_Shore	0.143322568	0.003631786	Hypermethylated
cg03551561	19		IGR	S_Shore	0.10316039	0.003687862	Hypermethylated
cg25817165	18	CNDP2	1stExon	S_Shelf	0.14821961	0.004003037	Hypermethylated
cg15652532	2	LCLAT1	TSS1500	N_Shore	0.229413098	0.004091997	Hypermethylated
cg21109666	1	DISC1	Body	opensea	0.144378264	0.004674427	Hypermethylated
cg06584561	13	LINC00545	TSS1500	opensea	0.108117117	0.005374395	Hypermethylated
cg10326673	2	LCLAT1	TSS1500	N_Shore	0.230589983	0.005428645	Hypermethylated
cg24432675	10	ADARB2	Body	N_Shore	-0.15503177	0.005700386	Hypomethylated
cg11323295	2		IGR	opensea	-0.114186522	0.007627878	Hypomethylated
cg20124410	13		IGR	opensea	0.138077396	0.008280267	Hypermethylated
cg04657146	19	HOOK2	Body	Island	0.222995686	0.008850185	Hypermethylated
cg06417478	19	HOOK2	Body	N_Shore	0.225331032	0.009043419	Hypermethylated
cg11738485	19	HOOK2	Body	Island	0.255586433	0.009071346	Hypermethylated
cg10095305	5	SKP2	5'UTR	opensea	-0.129279624	0.009438161	Hypomethylated
cg06211550	16		IGR	N_Shelf	0.11881646	0.009444653	Hypermethylated

cg02389040	6		IGR	Island	0.103058726	0.010421994	Hypermethylated
cg19553932	17		IGR	opensea	-0.111520186	0.010499868	Hypomethylated
cg10788750	13	RNF219-AS1	Body	opensea	0.106126161	0.011033108	Hypermethylated
cg23899408	19	HOOK2	Body	S_Shore	0.180419514	0.011641595	Hypermethylated
cg15881997	10	ADARB2	Body	opensea	-0.141225069	0.011918914	Hypomethylated
cg26033510	6		IGR	opensea	0.172757389	0.011927211	Hypermethylated
cg17468100	22	C22orf27	TSS200	N_Shore	0.101643719	0.013902448	Hypermethylated
cg19446385	12		IGR	opensea	-0.103026663	0.014703473	Hypomethylated
cg20082916	12	LOC100507065	Body	opensea	0.11078567	0.016679131	Hypermethylated
cg26446133	18	CNDP2	5'UTR	S_Shelf	0.105232824	0.017471628	Hypermethylated
cg06758191	4	AFAP1	Body	opensea	0.255473369	0.018512388	Hypermethylated
cg17836251	19	MYO9B	Body	opensea	-0.152675039	0.021068856	Hypomethylated
cg07253311	2	ATF2	Body	opensea	0.175491212	0.021351907	Hypermethylated
cg05510714	2	KYNU	Body	opensea	0.106351677	0.021856728	Hypermethylated
cg08219700	8		IGR	Island	0.114617048	0.023688796	Hypermethylated
cg12633154	6	RNF39	Body	Island	0.126974088	0.023840508	Hypermethylated
cg13294447	12	DIP2B	Body	opensea	0.116654493	0.024265887	Hypermethylated

cg26893861	17	DUSP3	3'UTR	opensea	0.138670415	0.02433385	Hypermethylated
cg18434912	6	AMD1	TSS1500	N_Shore	0.160137969	0.024746049	Hypermethylated
cg07694252	8	ANGPT1	Body	opensea	0.119786943	0.025071739	Hypermethylated
cg03028786	2	SERPINE2	5'UTR	opensea	0.237618555	0.026374273	Hypermethylated
cg01879420	6	AMD1	TSS1500	N_Shore	0.15586015	0.027153528	Hypermethylated
cg03245326	7		IGR	opensea	-0.101260495	0.027628137	Hypomethylated
cg05995465	2	HDAC4	5'UTR	opensea	-0.214628488	0.028511395	Hypomethylated
cg21685655	7	PON2	3'UTR	opensea	-0.10235891	0.02976975	Hypomethylated
cg01383724	9	LOC101929563	Body	opensea	0.149624959	0.030028711	Hypermethylated
cg17330251	7	PON1	TSS200	Island	-0.106171738	0.030487565	Hypomethylated
cg24078577	11	ASRGL1	3'UTR	opensea	-0.120253805	0.031234186	Hypomethylated
cg19472310	11		IGR	opensea	0.101130587	0.031653154	Hypermethylated
cg26370237	17	HSF5	Body	opensea	-0.136259539	0.032643838	Hypomethylated
cg11415067	5	MEGF10	5'UTR	opensea	-0.104704992	0.033292153	Hypomethylated
cg15961042	12		IGR	opensea	0.169584696	0.034007602	Hypermethylated
cg13835576	8	BLK	Body	opensea	-0.18745448	0.034397802	Hypomethylated
cg23248424	5	GFPT2	Body	Island	-0.147840702	0.034885611	Hypomethylated

cg10930308	6	RNF39	Body	Island	0.113411281	0.035351302	Hypermethylated
cg16078649	6	RNF39	Body	Island	0.100454507	0.03658063	Hypermethylated
cg19990651	6	HLA-DPB1	Body	Island	-0.101829862	0.037249202	Hypomethylated
cg13401893	6	RNF39	Body	Island	0.115545892	0.037292595	Hypermethylated
cg00483852	12	KCTD10	Body	opensea	-0.10199541	0.037472847	Hypomethylated
cg10318771	2	ADAM23	Body	opensea	0.104075051	0.038358467	Hypermethylated
cg23403192	21	USP16	TSS1500	N_Shore	-0.13412652	0.038906505	Hypomethylated
cg27586797	5		IGR	opensea	0.159090009	0.041271257	Hypermethylated
cg27049827	4		IGR	opensea	0.157758235	0.041970129	Hypermethylated
cg04553038	16	TLDC1	Body	opensea	0.10216764	0.042910026	Hypermethylated
cg22100363	1		IGR	opensea	0.101362181	0.043491975	Hypermethylated
cg11062466	8		IGR	N_Shore	0.109706934	0.045393755	Hypermethylated
cg02891314	5	GFPT2	Body	Island	-0.148804589	0.045814336	Hypomethylated
cg10864200	4	PCGF3	5'UTR	N_Shelf	-0.114438489	0.04591274	Hypomethylated
cg07613115	8	CCDC26	Body	opensea	0.10836686	0.048456332	Hypermethylated
cg03363289	9	LHX6	Body	Island	0.108976109	0.049975918	Hypermethylated
