# Supplementary material for: The prognostic value of whole-genome DNA methylation in response to Leflunomide in patients with Rheumatoid Arthritis
Source: Front Immunol. 2023 Sep 7;14:1173187. doi: 10.3389/fimmu.2023.1173187 (PMC10513488; doi:10.3389/fimmu.2023.1173187)
Supplement: Supplementary file 6 [file Table_4.pdf]

Supplementary Table 4.Comparison of parameters for constructing models using different machine learning methods

	Train						Test						Total					
	F1-score	Recall	Precision	Accuracy	Sensitivity	Specificity	F1-score	Recall	Precision	Accuracy	Sensitivity	Specificity	F1-score	Recall	Precision	Accuracy	Sensitivity	Specificity
RF	1.00	1.00	1.00	1.00	1.00	1.00	0.74	0.79	0.69	0.65	0.79	0.40	0.91	0.93	0.90	0.89	0.93	0.83
Adaboost	1.00	1.00	1.00	1.00	1.00	1.00	0.74	0.72	0.75	0.68	0.72	0.60	0.92	0.91	0.93	0.90	0.91	0.89
NB	0.75	0.80	0.70	0.67	0.80	0.48	0.73	0.77	0.70	0.65	0.77	0.44	0.74	0.79	0.70	0.67	0.79	0.41
SVM	0.97	1.00	0.95	0.97	1.00	0.92	0.76	0.84	0.69	0.66	0.84	0.36	0.90	0.95	0.86	0.88	0.95	0.76
Logistic	0.72	0.80	0.67	0.63	0.79	0.40	0.79	0.86	0.73	0.71	0.86	0.44	0.74	0.81	0.69	0.66	0.81	0.41

RF: random forest;SVM: Support Vector Machine; NB:naïve Baye
